# Sham surgery as an alternative procedure for unexplained pain following transvenous implantable cardioverter defibrillator implantation: case report

**DOI:** 10.1093/ehjcr/ytaf562

**Published:** 2025-11-13

**Authors:** Roin Rekvava, Nino Javakhidze, Elisabed Chikobava, Luka Kiguradze, Irakli Ninua

**Affiliations:** Department of Arrhythmology, American Hospital Tbilisi, Tbilisi 0159, Georgia; Department of Arrhythmology, American Hospital Tbilisi, Tbilisi 0159, Georgia; Faculty of Medicine, David Tvildiani Medical University, Tbilisi 0159, Georgia; Department of Arrhythmology, American Hospital Tbilisi, Tbilisi 0159, Georgia; Faculty of Medicine, American MD Program, Tbilisi State Medical University, Tbilisi 0186, Georgia; Department of Arrhythmology, American Hospital Tbilisi, Tbilisi 0159, Georgia; Faculty of Medicine, David Tvildiani Medical University, Tbilisi 0159, Georgia; Department of Arrhythmology, American Hospital Tbilisi, Tbilisi 0159, Georgia

**Keywords:** ICD implantation, Cardiac implantable electronic devices (CIED), Sham surgery, Psychogenic pain, Sudden cardiac death

## Abstract

**Background:**

Persistent pain after implantable cardioverter-defibrillator (ICD) implantation is uncommon and often unexplained after ruling out device-related or musculoskeletal causes. Psychological factors can contribute to symptom persistence, complicating treatment. In rare cases, sham surgery has been considered to address psychogenic pain. This report explores its role in managing atypical post-ICD implantation pain.

**Case summary:**

A 76-year-old female with hypertrophic cardiomyopathy, atrial fibrillation, and a history of ICD implantation presented with persistent left parasternal pain radiating to the arm and back. Extensive evaluations ruled out device-related complications, musculoskeletal issues, and nerve injury. Psychological assessment suggested a psychogenic origin, likely reinforced by social factors. After ethics approval, sham surgery was performed, leading to complete symptom resolution and improved quality of life. This case highlights the potential role of psychological interventions in managing medically unexplained post-ICD implantation pain.

**Discussion:**

Post-ICD implantation pain ranges from routine discomfort to complications requiring intervention. Causes include device migration, musculoskeletal issues, nerve damage, infections, and psychological factors. Thorough evaluation is essential to rule out organic causes. Management includes non-steroidal anti-inflammatory drugs, physical therapy, neuropathic pain medications, and psychological support. This case highlights the role of psychogenic factors in persistent pain.

Learning points

- Importance of distinguishing between physical and psychological causes of postoperative pain following implantable cardioverter-defibrillator (ICD) implantation is essential for appropriate treatment.
- A multidisciplinary approach, including cardiology, neurology, orthopaedics, and psychiatry, is crucial in the comprehensive evaluation of unexplained pain.
- Psychological factors: anxiety and attention-seeking behaviours can significantly contribute to the perception of pain and should be addressed in the management of complex post-surgical symptoms.

## Introduction

Implantable cardioverter-defibrillators (ICDs) are life-saving devices commonly used for the primary or secondary prevention of sudden cardiac death, particularly in patients with ventricular arrhythmias or in conditions associated with high arrhythmic risk such as hypertrophic cardiomyopathy (HCM). In this case, the patient received the ICD for primary prevention of sudden cardiac death in the context of HCM, consistent with guideline recommendations.^1^

However, in rare cases, patients may develop atypical, unexplained pain after device implantation, which can have significant physical and psychological impact. When all identifiable causes of pain, such as device malfunction or nerve damage, are excluded, treatment options become limited. Psychological contributors and behavioural reinforcement can maintain symptoms in some patients.^2^ Sham procedures and placebo effects have been described in procedural literature and in placebo research, and they may be relevant when purely medical causes are excluded.^3^ Recent articles describe device-related migration and non-cardiac mimics of chest pain after cardiac procedures.^4–6^

## Summary figure

**Figure:**
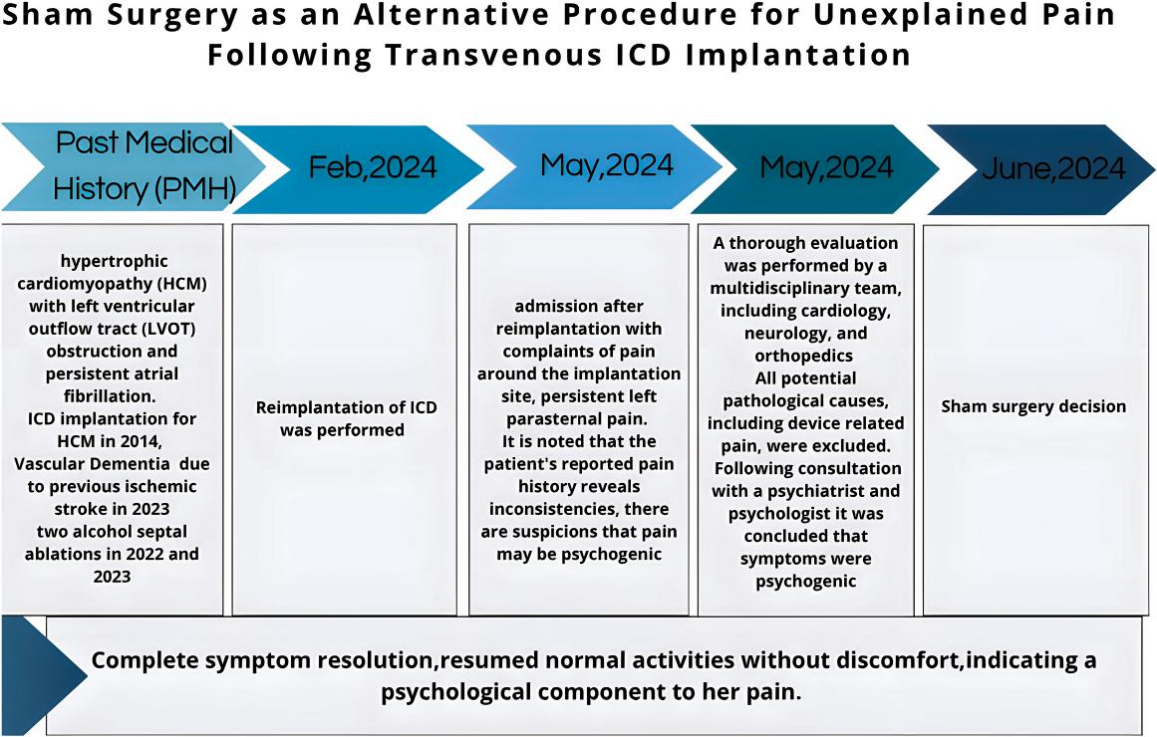


## Case presentation

A 76-year-old female with a complex cardiovascular history presented with left parasternal and subclavicular pain radiating to the left arm and scapular area. The pain was dull and persistent with episodic intensification. It was associated with a restricted range of motion in the left upper limb, especially on abduction, and with discomfort around the ICD implantation site. The symptoms started approximately 3 months after generator replacement in February 2024; the patient presented in May 2024.

In February 2024, the patient underwent an upgrade from a single-chamber to a dual-chamber ICD system. Initial procedure involved the replacement of a previously implanted single-chamber ICD with a dual-chamber system. During this intervention, a new atrial lead was implanted to enable dual-chamber pacing, while the pre-existing ventricular lead was retained without modification.

Her past medical history included HCM with left ventricular outflow tract (LVOT) obstruction and persistent atrial fibrillation. The patient had been under regular cardiology care since her first ICD implantation for HCM in 2014. Because of severe LVOT obstruction, she underwent two alcohol septal ablations in 2022 and 2023, which reduced the interventricular septum (IVS) thickness from 26 to 16 mm and decreased the LVOT gradient from 90 to 30 mmHg. She had multiple heart failure rehospitalizations and prior pleural cavity drainage before the ablations.

During admission, her most recent cardiac evaluation showed mildly reduced left ventricular function with an ejection fraction (EF) of 47%. Echocardiography demonstrated systolic anterior motion (SAM) of the mitral valve, LVOT gradient 30 mmHg, moderate mitral and tricuspid regurgitation, mild aortic regurgitation, and pulmonary artery systolic pressure (PASP) of 60 mmHg. Coronary angiography showed no significant coronary artery disease. Her medication regimen included rivaroxaban 20 mg, metoprolol, and levothyroxine for hypothyroidism, following a history of iatrogenic thyrotoxicosis post-thyroidectomy for multinodular toxic goitre.

The patient lives in a retirement home; her children live abroad. Because of vascular dementia affecting decision-making capacity, her son served as her designated decision-maker.

During the present admission, she reported persistent left parasternal pain that required further workup. Caregivers noted that she does not spontaneously report pain unless directly asked. A multidisciplinary team (cardiology, neurology, orthopaedics) performed a thorough evaluation.

Serial inflammatory markers and C-reactive protein (CRP) and erythrocyte sedimentation rate (ESR) remained within normal limits. On examination, there were no signs of venous congestion. Shoulder radiography showed no abnormalities. Shoulder ultrasound revealed no effusion, tendon tear, or bursitis. Peripheral nerve ultrasound showed no nerve entrapment or neuroma. Clinical neurological examination revealed no motor or sensory deficit. Nerve conduction studies were normal, effectively excluding neuropathic aetiology. These evaluations revealed no significant pathology.

All pathological causes, including device malfunction, were ruled out, with lab results, cardiac ultrasonography, and echocardiography showing no progression of underlying conditions. A contralateral device repositioning was planned to alleviate the patient’s symptoms, but after scheduling, the patient stopped reporting pain. Following consultation with a psychiatrist and psychologist, it was noted that individuals experiencing pain often receive attention, care, and affection from family and friends, which can socially reinforce pain-related behaviours. Based on evaluation, it was concluded that symptoms were psychogenic, likely driven by anxiety and attention-seeking behaviour reinforced by family interactions.

Following institutional clinical ethics committee approval and informed consent from the patient’s son (the legal representative), the team performed a sham procedure. The ethics committee included medical, legal, and patient-care representatives and concluded the intervention was ethically defensible given the patient’s diminished capacity, persistent distress, and the absence of viable medical alternatives.

The sham procedure was performed under local anaesthesia using a minimally invasive approach. A small incision was made below the left clavicle near the implantation site. The patient was told that the device had been repositioned to relieve symptoms. Routine wound care and follow-up were provided to support the placebo effect within an ethical framework.

Post-procedural follow-up was conducted immediately after the surgery for 1 week, with evaluations every 2 days to ensure the effectiveness of the sham intervention. Thereafter, the patient has been scheduled for routine follow-up visits every 3 months for ICD assessment.

After the sham procedure, the patient and her family reported complete symptom resolution, including retrosternal pain and mobility limitation. She resumed normal activities without discomfort and reported improved quality of life. A psychologist observed that the placebo surgery likely increased the patient’s receptiveness to recovery, reduced anxiety, and improved pain perception, leading to symptomatic relief.

## Discussion

Postoperative pain after ICD implantation is common and typically occurs in the chest, parasternal, or shoulder regions. Understanding the underlying causes is critical for accurate diagnosis and effective treatment. Pain may range from expected post-surgical soreness to more complex conditions requiring intervention. Early pain often reflects the inflammatory response to tissue trauma from incision or device placement; persistent or worsening pain beyond expected recovery requires further investigation.^1^

Device-related complications must be considered for persistent pain after ICD implantation. Improper placement, migration, or lead perforation can lead to local tissue irritation or nerve injury. Imaging (X-ray, fluoroscopy, or targeted computed tomography) can confirm lead and device position. If complications are confirmed, surgical revision may be required. Device migration and lead perforation can present as persistent or acute chest pain. Researchers have described implantable loop recorder migration causing chronic pleuritic pain and haemothorax after device implantation.^4,5^

Musculoskeletal causes such as rotator cuff injury or adhesive capsulitis may result from procedural trauma or immobilization and can present as shoulder or parasternal pain. Diagnosis relies on clinical examination and imaging; management includes physical therapy, corticosteroid injections, or surgery when indicated.

Neuropathic pain from intraoperative nerve injury or neuroma formation may cause sharp, radiating pain to the shoulder and arm. Treatment options include neuropathic agents (e.g. gabapentinoids), nerve blocks, or neuromodulation for refractory cases.

Psychological factors can amplify or perpetuate pain after device procedures. Anxiety about the device, fear of shocks, and hypervigilance can worsen symptom perception and functional impairment. Behavioural reinforcement from caregivers may also prolong symptom duration. Structured psychological interventions and education can reduce distress and improve outcomes.^2^

Procedural stress and non-cardiac pathology may produce persistent chest symptoms and functional presentations. Careful, stepwise diagnostic evaluation is advised to avoid unnecessary invasive interventions and to identify non-cardiac causes.^6^

Infection at the implantation site must also be excluded. Prompt antibiotic therapy and, in severe cases, device removal or revision may be required.

Pain management after device implantation includes non-steroidal anti-inflammatory drugs (NSAIDs) for inflammation, physical therapy for musculoskeletal causes, and neuropathic agents for nerve pain. Psychological support is a key adjunct and may reduce the need for further invasive treatment.

Sham procedures have traditionally been employed in clinical trials to evaluate the efficacy of surgical or interventional treatments by controlling for placebo effects. Notable examples include the SHAM-PVI study and the ORBITA-AF trial, which investigated the placebo effect in atrial fibrillation ablation procedures. These trials underscore the powerful therapeutic impact of procedural context, even when no therapeutic intervention is performed. However, the use of sham surgery in clinical—not investigational—settings remains rare and ethically contentious. In our case, the sham procedure was not part of a research protocol but was instead used therapeutically under clinical ethics oversight. While research settings demand rigorous consent and blinding protocols, the clinical use necessitates individualized ethical justification centred on patient welfare, capacity, and lack of viable alternatives.

This case raises important ethical considerations surrounding deception and informed consent in patients lacking capacity. In this scenario, the patient’s legal representative, her son, was fully informed, and the ethics committee endorsed the intervention as being in the patient’s best interest, given the lack of alternatives and substantial suffering. In patients with capacity, however, the use of sham procedures would necessitate a different ethical approach. Some frameworks suggest that sham procedures may be ethically permissible under conditions of enhanced informed consent, where the placebo nature is disclosed but the potential psychological benefit is explained. Others argue that true informed consent inherently conflicts with the therapeutic value of deception. Therefore, sham procedures in capacitated patients would require individualized consideration, potentially under research protocols or well-justified clinical ethics review.

## Conclusion

This case highlights the role of sham surgery and psychological interventions in managing medically unexplained symptoms. While sham surgery is not standard, its use in carefully selected cases where psychogenic factors are strongly suspected can offer relief, provided that ethical considerations are addressed. The patient or their legal representative is fully informed.

The decision to proceed with the sham surgery was made with careful consideration of multiple factors. The potential benefits in alleviating the patient’s distress and improving her quality of life were weighed against the risks, especially given that no other interventions had provided relief, and all pathological causes had been ruled out.

The success of the sham surgery in this case emphasizes the importance of recognizing psychogenic pain in patients with complex medical histories, particularly in those undergoing repeated or invasive procedures. Early identification and management of such pain can prevent unnecessary surgical interventions and improve patient outcomes.

In conclusion, this case demonstrates that sham surgery may be a viable option for managing atypical, device-induced pain when psychological factors are suspected. While it should not be routinely performed, in select cases where all other possibilities have been ruled out, it can significantly improve patient quality of life.

## Data Availability

No new data were generated or analysed in support of this research.

## Lead author biography

Roin Rekvava, a cardiologist and heart rhythm specialist, is the Head of the Heart Rhythm Management and Electrophysiology Department at American Hospital Tbilisi (AHT). He is also an Associate Professor at Caucasus International University, a member of the Arrhythmia Council of the Asian Heart Society, and Vice President at SRHRS.

## Notes

**Consent:** The authors confirm that written consent for the submission and publication of this case report has been obtained from the patient in accordance with the COPE guidelines.

**Funding:** This case report did not receive any specific grant from funding agencies in the public, commercial, or not-for-profit sectors.
